# Molecular Assembly and Structural Plasticity of Sensory Ribbon Synapses—A Presynaptic Perspective

**DOI:** 10.3390/ijms21228758

**Published:** 2020-11-19

**Authors:** Roos Anouk Voorn, Christian Vogl

**Affiliations:** 1 Presynaptogenesis and Intracellular Transport in Hair Cells Junior Research Group, Institute for Auditory Neuroscience and InnerEarLab, University Medical Center Goettingen, 37075 Goettingen, Germany; roosanouk.voorn@med.uni-goettingen.de; 2 Göttingen Graduate Center for Neurosciences, Biophysics and Molecular Biosciences, 37075 Goettingen, Germany; 3 Collaborative Research Center 889 “Cellular Mechanisms of Sensory Processing”, 37075 Goettingen, Germany

**Keywords:** peripheral auditory pathway, synaptic sound encoding, cochlear development, synapse maturation, cytoskeleton, molecular motors

## Abstract

In the mammalian cochlea, specialized ribbon-type synapses between sensory inner hair cells (IHCs) and postsynaptic spiral ganglion neurons ensure the temporal precision and indefatigability of synaptic sound encoding. These high-through-put synapses are presynaptically characterized by an electron-dense projection—the synaptic ribbon—which provides structural scaffolding and tethers a large pool of synaptic vesicles. While advances have been made in recent years in deciphering the molecular anatomy and function of these specialized active zones, the developmental assembly of this presynaptic interaction hub remains largely elusive. In this review, we discuss the dynamic nature of IHC (pre-) synaptogenesis and highlight molecular key players as well as the transport pathways underlying this process. Since developmental assembly appears to be a highly dynamic process, we further ask if this structural plasticity might be maintained into adulthood, how this may influence the functional properties of a given IHC synapse and how such plasticity could be regulated on the molecular level. To do so, we take a closer look at other ribbon-bearing systems, such as retinal photoreceptors and pinealocytes and aim to infer conserved mechanisms that may mediate these phenomena.

## 1. Introduction

**Figure 1:**
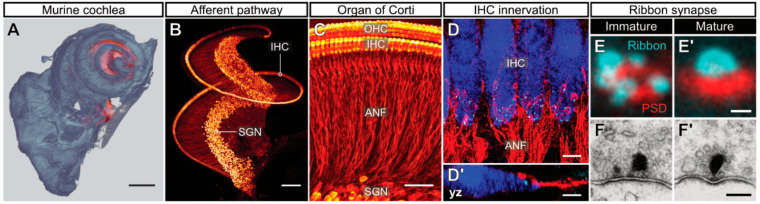
Structural overview of the peripheral auditory pathway. Light-sheet microscopic 3D- reconstructions of (**A**) the bony murine cochlea that harbors the organ of Corti and (**B**) the isolated peripheral auditory system, with afferent spiral ganglion neurons (SGNs) branching out towards the row of inner hair cells (IHCs), in the typical spiraling staircase anatomy. IHCs and SGNs are labeled with an antibody against the cytosolic Ca^2+^-buffer Calretinin. (**C**) Confocal maximum projection of the organ of Corti labeled for the cytosolic Ca^2+^-buffer Parvalbumin, displaying the three rows of outer hair cells (OHCs), the single row of IHCs and the afferent innervation (auditory nerve fibers; ANF) by the SGNs. (**D**) Innervation of IHCs (Calbindin; blue) by individual SGN neurites (a3-Na^+^/K^+^-ATPase; red), showing the presynaptic ribbons (CtBP2; cyan) in contact with the postsynaptic SGN boutons. (**D’**) Side view of the innervated IHC showing the basolateral position of the synaptic ribbon and connected bouton. (**E**,**E’**) STED microscopic images of IHC synaptic ultrastructure of (**E**) a developing IHC active zone distributed in several precursor spheres colocalizing with multiple clusters of the postsynaptic density (PSD) versus (**E’**) one large ribbon opposing one ellipsoid PSD in a mature preparation. (**F**,**F’**) Electron microscopic images of (**F**) immature multi-ribbon active zones with roundish profiles versus (**F’**) the wedge-shape of a mature IHC ribbon that is attached to the curved presynaptic membrane. Scale bars: A 300 µm; B 150 µm; C 50 µm; D-D’ 5 µm; E-E’ 250 nm; F-F’ 200 nm. (**B**,**E**,**E’**) with permission from Reference [4]; (**D**) with permission from Reference [5].

Hearing relies on the ultrafast, temporally-precise and frequency-specific encoding of sound into neural signals. In mammals, this process takes place in the cochlea, a snail-shaped structure deeply embedded within the temporal bone (Figure 1A). The cochlea harbors the highly structured sensory epithelium—the organ of Corti—which consists of three rows of electromotile outer hair cells (OHCs) and one row of sensory inner hair cells (IHCs) that are organized in a tonotopic manner (Figure 1B,C). While OHCs play a key role in active cochlear amplification, IHCs are the true sensory receptor cells that perform the challenging task of synaptic sound encoding, that is, the transformation of a physical stimulus into neural code at their ribbon synapses with postsynaptic spiral ganglion neurons (SGNs). To adequately fulfill this task, IHCs form between 5–30 monosynaptic connections with individual type I SGNs (Figure 1D,D’), which make up ~95% of the spiral ganglion, whereas the remaining ~5% of type II SGNs form *en passant* connections with multiple OHCs [1,2,3]. IHC presynaptic active zones (AZs) are anatomically distinct from conventional neuronal synapses, as they are characterized by electron-dense projections at their presynaptic membranes—so-called synaptic ribbons—which tether a halo of synaptic vesicles (SVs). Auditory ribbon-type synapses play an essential role in the ultrafast and temporally-precise neurotransmission and undergo substantial developmental refinement prior to hearing onset (Figure 1E–F’), ultimately enabling hearing with a high dynamic range that spans several orders of magnitude in sound intensity.

**Figure 2:**
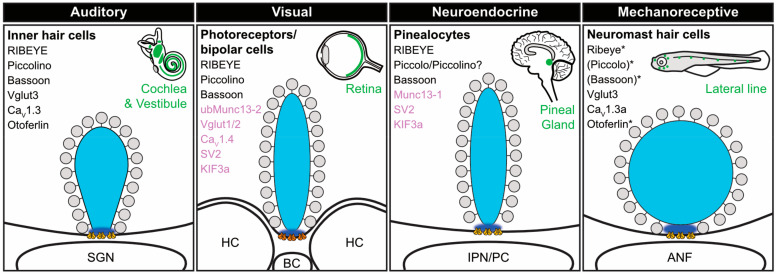
Ribbon synapse morphology and molecular composition differ between biological systems. Schematic drawings of stereotypic ribbon shapes from the indicated sensory or neuroendocrine system they are operating in, to illustrate gross morphological and molecular differences: Selected conserved (black) and non-conserved (magenta) ribbon-associated proteins are highlighted in the respective boxes. Insets show the approximate locations of the ribbon-bearing cell populations (green) in a larger context. Please note: Ribbon dimensions are not drawn to absolute scale. Zebrafish gene duplications are highlighted with an *, Piccolo a/b and Bassoon b are expressed on mRNA level (https://piotrowskilab.shinyapps.io/neuromast_homeostasis_scrnaseq_2018); however, protein localization to the ribbon remains to be demonstrated. ANF, afferent nerve fiber; BC, bipolar cell; HC, horizontal cell; IPN, intrapineal neuron; PC, pinealocyte; SGN, spiral ganglion neuron.

Synaptic ribbons are not exclusive to IHCs, but also found at presynaptic AZs of other post-mitotic sensory receptor cells as well as selected neuronal populations—such as mammalian cochlear OHCs, vestibular hair cells, retinal photoreceptors and bipolar cells, pinealocytes as well as avian inner ear hair cells and fish inner ear and lateral line neuromast hair cells—where they serve essential functions in AZ scaffolding and presynaptic SV release and replenishment. Independent of the sensory system they are operating in, the main molecular component of synaptic ribbons is the evolutionary conserved protein RIBEYE [6,7], which forms the structural backbone of the scaffold and clusters voltage-dependent Ca^2+^ channels (Ca_V_s) in the presynaptic plasma membrane at its base [8,9]. Ribbons across different species and cell types show a high level of morphological and molecular similarity and yet, are specialized according to the particular need of the respective biological system (Figure 2; [10,11,12]) and differ in their spatial dimensions and specific shapes (Table 1). This is important to keep in mind when comparing experimental findings from different ribbon populations and attempting to draw conceptual conclusions. Yet, a unifying concept of all ribbon types is the structural stabilization of essential AZ components at the presynaptic membrane—such as Ca_V_s and so forth—during bouts of prolonged and extensive membrane turnover.

In mammals, ribbon-bearing sensory receptor cells—such as IHCs and photoreceptors—cannot be regenerated once lost and hence, are essentially required to encode sensory information flawlessly across the entire life span of an organism. While the same principle holds true for virtually all neurons in the brain, IHCs in particular are strongly limited in number—a mouse cochlea harbors only ~700–800 sensory IHCs; [13])—and thus, IHC loss or synaptic dysfunction cannot easily be compensated on systems level. Moreover, IHC synapses are required to restlessly decompose sound into neural code with utmost temporal precision and extraordinarily high SV release rates (i.e., in mice, SGN instantaneous spike rates can exceed 1 kHz at sound onset, while adapted rates plateau ~300 Hz; [14]). Such functional demands require a significant level of long-term structural stability—but likely also adaptive plasticity—of the synaptic complex. Over recent years, deciphering the molecular anatomy and cellular function of the ribbon in presynaptic release has been a major focus of sensory systems research (recently reviewed in Reference [10]). Despite these efforts, its functional role is still unclear; however, various hypotheses have been put forward. These include a role in SV recruitment, priming, replenishment and reformation [8,15,16,17,18,19,20,21,22,23] as well as the scaffolding of presynaptic Ca^2+^ channels and release sites [8] and potentially the formation of a physical Ca^2+^ diffusion barrier at the AZ [24]. While the knowledge of the functional role and molecular anatomy of mature IHC ribbons is ever expanding, information on the early assembly and developmental maturation of auditory ribbon-type AZs prior to hearing onset (~p12 in mice; [25]) remains comparably scarce. Similarly, despite of their functional implications, other important physiological processes—such as structural plasticity—are still elusive to date, largely due to the technical difficulties in simultaneously visualizing, while adequately stimulating, the tissue in vivo and even in vitro. 

In the following, we aim to briefly summarize the current state of knowledge regarding auditory ribbon synapse assembly and structural plasticity. For this purpose, while primarily focusing on auditory IHC ribbons, we will also integrate available information from other ribbon-type synapses to infer likely common principles. Further, we aim to retain a clear focus on the presynaptic AZ and discuss (i) afferent synaptogenesis, (ii) the molecular composition and cellular origin of ribbon precursors, (iii) early AZ assembly and its molecular, structural and functional maturation, (iv) structural plasticity of ribbon synapses and the role of synaptic activity in this process and finally, (v) take a closer look at cytoskeletal components likely mediating precursor transport and ribbon plasticity. Since this scope is limited to the presynapse, we would like to point the interested reader towards more exhaustive review articles focusing on hair cell and general afferent synapse development [37,38,39,40], spontaneous activity in the immature organ of Corti [41] and auditory ribbon synapse anatomy as well as (patho-)physiology [5,42,43].

## 2. Developmental Assembly of Auditory Ribbon Synapses

**Figure 3:**
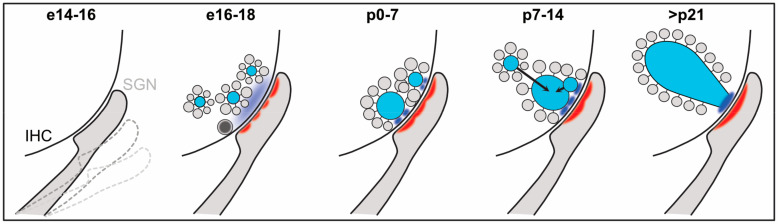
Synaptogenesis in murine cochlear IHCs. The formation of the synaptic connection between inner hair cells (IHCs) and spiral ganglion neurons (SGNs) starts in the late embryonic stages. Afferent contact formation precedes the arrival of pre- and postsynaptic proteins. In the late prenatal stages, ribbon precursors (cyan) are formed in the IHC cytoplasm and already tether synaptic vesicles (SVs). The precursors translocate to the developing active zone (AZ) within the basolateral compartment of the IHC. Dense core vesicles (black and gray), may play a role in AZ-directed transport of proteins not localizing to the precursors. In early postnatal stages ribbon precursors attach at the presynaptic membrane, where they fuse alongside their individual presynaptic densities (dark blue). Subsequently, ribbons grow and the size of the attached SVs decreases, while their number increases until hearing onset. Simultaneously, the postsynaptic densities convert from multi-cluster organization to one ellipsoid density per bouton. Developmental refinement of both pre- and postsynapse results in the innervation of predominantly one AZ per SGN and—in the majority of synapses—one single large, droplet-shaped and mature ribbon per AZ.

**Figure 4:**
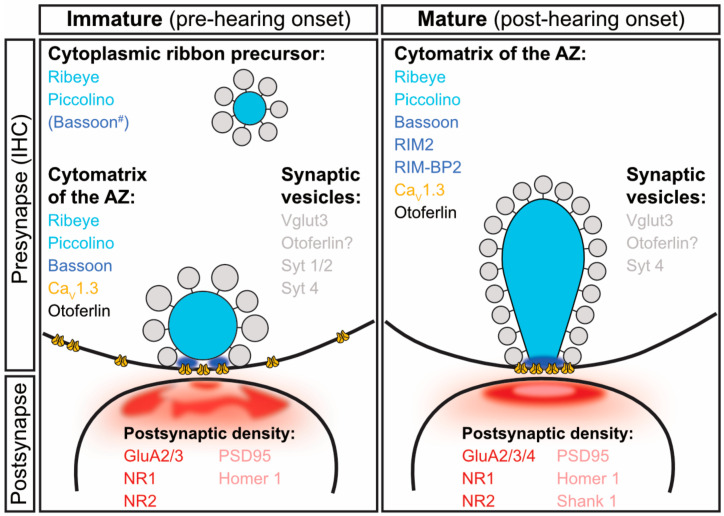
Molecular maturation of ribbon-type AZs. Molecular composition of a murine ribbon synapse between inner hair cells (IHCs) and spiral ganglion neurons (SGNs) and its differential constitution during early postnatal development (prior to hearing onset) and in matured stages (after hearing onset). Along with the growth, membrane attachment and fusion of precursors, the establishment of a mature ribbon synapse involves the spatial confinement of its molecular components: Ca_V_s localize in tight clusters underneath the ribbon, multiple presynaptic densities merge to one single ribbon anchor and postsynaptically one continuous elongated postsynaptic density is formed that stabilizes a ring-like organization of AMPA receptors. A developmental switch in the regulation of presynaptic exocytosis is present in the downregulation of Synaptotagmin (Syt) 1 and 2, which are likely absent from mature ribbons. Here, the IHC-specific multi-C2-domain protein Otoferlin becomes responsible for the regulation of synaptic vesicle release in the early post-natal stages, despite expression from embryonic ages. (#) Bassoon is part of ribbon precursor spheres in the retina. The here illustrated divergence in the illustrated protein expression between immature and mature preparations does not necessarily reflect absence of expression in the young tissue *per se* but rather results from the current lack of experimental evidence from pre-hearing preparations. For more detailed information please refer to the respective parts in the text. Font color indicates association with the correspondingly colored pre-/postsynaptic compartment.

In mice, depending on their tonotopic position, IHCs differentiate at the beginning of the third embryonic week with a base-to-apex developmental gradient [44]. Rapidly after these cells emerge, as early as embryonic day (e)16.5, voltage-dependent Ca^2+^ currents as well as exocytic activity—measured as vesicular fusion in response to IHC depolarization—can already be detected [45]. This early release capability temporally coincides with the establishment of early synaptic contact sites between IHCs and invading SGN neurites [4]. Subsequently, these afferent connections undergo extensive morphological and functional maturation to become synaptically active entities. For example, SGN collateral arborizations are pruned during maturational refinement that ultimately establishes the characteristic monosynaptic connections [46,47,48,49], in a process that strongly depends on thyroid hormone signaling [50,51,52]. In parallel, particularly during late embryonic to early postnatal development, the assembly and maturation of individual IHC ribbon-type AZs is comprised of a dynamic shaping and re-shaping process, which finalizes around hearing onset in mice (Figure 3; [4,27,39,46,53,54,55]). Initially, ribbon precursors assemble in the cytoplasm via RIBEYE self-aggregation to form free-floating precursors that are spherical in shape and have been shown to tether a set of immature SVs. This mechanism is highly conserved across all ribbon-bearing systems [4,53,56,57] and could even be observed after exogenous expression of RIBEYE in commonly used cell lines [33,58]. This observation indicates that cytoplasmic ribbon precursors are likely not pure RIBEYE aggregates but rather suggestive of multi-protein organelles that also contain other cytomatrix proteins and can hence be considered as presynaptic “building blocks.” Over the course of development, these free-floating precursors grow in size and relocate to the presynaptic membrane, where they either (i) attach at the AZ plasma membrane, (ii) fuse with each other in close proximity to the AZ membrane or (iii) fuse with already membrane-anchored ribbons [4,53,54]. Once membrane-anchored, ribbons further expand in size until the end of the second postnatal week in mice, whereupon ribbon volume has reached its maximum extent and appears to remain largely stable thereafter [4]. During this process, the ribbon-bound SV pool increases with expanding ribbon volume. Interestingly, ribbon-attached SVs appear to mature in parallel to the scaffold with a comparable time course and shrink in diameter, which ultimately enables an even greater SV packing density on mature ribbons. These latter findings suggest that ribbons form a multi-functional scaffold that—apart from its established roles in structural AZ stabilization, SV priming and replenishment—may also facilitate certain aspects of developmental maturation. Whether such an involvement is of a direct or indirect nature, for example via other AZ proteins, remains to be determined.

Consistent with a proposed function of the ribbon as a conveyor belt resupplying fusion-competent SVs to the release site, recent live-cell imaging experiments on retinal bipolar cells suggest that SV movement along the ribbon is primarily unidirectional in close proximity to the presynaptic membrane [59,60]. Due to the high frequency of SV exocytosis occurring at sensory ribbon synapses, mere diffusion could be assumed to be insufficient to support timely SV replenishment. However, a recent modelling study proposed an efficient and rapid diffusion mechanism in which the filamentous tethers emanating from the ribbon surface facilitate a targeted SV flow towards the presynaptic membrane via rapid binding/unbinding of multiple tethers and proximal SVs [32]. To date, the molecular nature of these tethers remains unknown but its identification is an important target of current research [61]. As an alternative to the passive diffusion hypothesis of ribbon-attached SVs, previous studies in the retina and pineal gland have implicated molecular motors—such as the Kinesin Kif3a—as potential candidate molecules actively driving SV translocation to the release site [11,62]. However, this hypothesis remains controversial in the field and currently no data supporting such a mechanism at IHC AZs has been reported; thus, the driving factor in SV dynamics remains to be identified.

### 2.1. Lessons Learned from RIBEYE Mutants

Since the ribbon plays a central and facilitatory role in SV exocytosis and replenishment, we will briefly summarize recent work from RIBEYE mutants, before then taking a closer look at the putative functional roles and developmental assembly of the other molecular constituents of IHC AZs. 

#### 2.1.1. Effects of Ribeye Manipulation on Zebrafish Lateral Line Neuromast Hair Cells

In zebrafish, morpholino knock-down (KD) of *ribeye a* [63,64] and/or *ribeye b* [9] led to ribbon loss, impaired exocytosis and subsequent loss of postsynaptic contacts. A separate approach that employed zinc finger nucleases and CRISPR/Cas9 to generate loss-of-function mutations in *ribeye a* and *b* respectively, resulted in the formation of smaller, mislocalized and translucent “ghost ribbons” [65]. These latter abnormal ribbons retained the ability to tether a halo of SVs—even with a higher packing density than wild-type ribbons—indicating that the low remaining amounts of Ribeye in these mutants were sufficient to establish a multi-protein complex. Surprisingly, mutant hair cells showed larger Ca^2+^-currents and concomitant depolarization-induced exocytosis, which appeared to result from increased expression and redistribution of presynaptic Ca_V_s. Yet, these structural disturbances did not manifest in altered afferent responses [65]. Interestingly, in the same model system, previous Ribeye overexpression experiments reported that the induced enlargement of individual ribbons and overall increase in ribbon counts per hair cell similarly failed to affect exocytosis [35]. In both cases, homeostatic plasticity mechanisms at the postsynaptic side may compensate the presynaptic phenotype.

#### 2.1.2. RIBEYE Deletion in Mice Leads to Extensive Remodeling of the AZ

As expected, genetic deletion of RIBEYE in mice causes ribbon loss, disruption of the AZ morphology as well as a reduction of SVs at the AZ [17,18,19,66,67]. However, the observed functional deficits—at least in the auditory system—were rather mild. For example, capacitance measurements of murine *Ribeye*-knock-out (KO) IHCs displayed only a mild attenuation of exocytic performance [18,19]. Yet, synaptic release was found to be less accurate than in wild-type littermates and led to an impaired temporal precision of sound encoding. A much more severe functional phenotype could be observed in retinal bipolar cells, where *Ribeye*-KO caused a strong reduction in the readily-releasable pool (RRP) of SVs, plus a slight decrease in sustained transmission between bipolar and AII amacrine cells [17].

Apart from assisting in clarifying the functional role of the ribbon, the constitutive *Ribeye*-KO mouse model also offers deeper insights into the consequences of the physical absence of the ribbon structure itself. For example, previous work proposed that the ribbon scaffold—apart from SV replenishment—may act as a Ca^2+^ diffusion barrier that ensures highly localized Ca^2+^ maxima at the release sites [21,24,68]. Indeed, upon ribbon depletion, an increased size of Ca^2+^ spread could be detected in IHCs after activation [18] and mathematical modelling of the Ca^2+^ diffusion barrier hypothesis for frog saccular hair cells suggests that the presence of the ribbon is able to significantly increase the local Ca^2+^ concentration at the AZ [24]. Consistent with this hypothesis, recent data from combined electrophysiological and live-cell super-resolution Ca^2+^-imaging experiments on IHCs of *Bassoon* mutants—that lack membrane-anchored ribbons at their AZs—showed a larger and more diffuse Ca^2+^ spread than wildtype AZs upon stimulation [69]. While these findings are seemingly well compatible with a spatially tightly limited Ca^2+^ spread at the foot of the ribbon complex, other observations also need to be taken into consideration: in *Bassoon* mutants, the Ca_V_ distribution at the AZ is much less spatially confined than in wildtype IHCs [8]. Similarly, the presynaptic contacts of *Ribeye*-KO IHCs were shown to be decorated with multiple small ribbonless AZs with complex spatial configurations. Therefore, these findings offer an alternative explanation for the observed broader presynaptic Ca^2+^ spread in these mutants [18]. Thus, it is currently difficult to dissect the actual contribution of the ribbon as a physical Ca^2+^ diffusion barrier at this stage.

In summary, while the exact role of the ribbon in presynaptic release still remains largely unresolved, the analyses of the different *Ribeye* mutants has provided important insights. Even more so, these mutants have taught us one other—equally important—lesson: elaborate compensatory mechanisms re-shape the presynaptic architecture during developmental maturation to ensure maintained functionality of the afferent IHC synapse. For example, as mentioned above, IHC AZs of constitutive *Ribeye*-KO mice are characterized by fragmented multi-AZ presynaptic contacts that thereby recruit a sufficiently large RRP to sustain near-normal neurotransmission [18,19]. Given these findings, future analysis of a cell type-specific, inducible *Ribeye*-KO mouse model would be desirable to clarify ribbon function once the developmental assembly process has been completed, for example via genetic disruption subsequent to presynapse formation. In addition, other experimental strategies that acutely disrupt ribbon function via photodamage—such as fluorophore-assisted light inactivation (FALI) [15,70,71]—should be more widely employed across sensory systems to gain further insights into the functional role of the ribbon. 

### 2.2. Developmental Assembly of Other Essential AZ Proteins

While the ribbon dominates the presynaptic landscape from early postnatal ages, it also crucially depends on the presence of other AZ proteins, for example to provide anchor points at the presynaptic membrane for precursor attachment. Especially during the late embryonic stages, when afferent contacts have already been established, clearly demarcated pre- and postsynaptic densities can often be identified even prior to ribbon arrival at the AZ [4], but also see [54]. These findings are indicative of an early proteinaceous cytomatrix at the AZ of, so far, largely unknown nature. In photoreceptors, ribbon precursor spheres appear to act as vehicles for other cytomatrix proteins—including Bassoon, Piccolino and CtBP1 [57,72,73,74] and the same can also be expected to hold true for auditory IHCs. However, to date—apart from RIBEYE—only Piccolino could be identified as an integral part of cytoplasmically free-floating ribbon precursors in developing IHCs (Figure 4; [4]). Apart from the ribbon itself, multiple other AZ components have been shown to play essential roles in adult ribbon synapse function as will be outlined below. However, for the majority of these it remains unclear (i) at what stage during developmental maturation they arrive at the AZ, (ii) how they are trafficked to the AZ and (iii) how/when the individual AZ confinement is finalized and their mature distribution pattern established. In this context, dense-core vesicles (DCVs), which can also be observed at developing IHC AZs [4,54], may contribute to initial synapse assembly. Their transient and sparse appearance during late embryonic to early postnatal stages fits the timeline of AZ differentiation prior to ribbon arrival and may indicate a function in transporting other AZ components to the presynapse; yet, their exact role—if any—in this process remains largely unclear to date. Alternatively, DCVs may serve an entirely different purpose in the storage and release of neurotrophic factors, in order to stimulate and maintain the synaptic connections with SGNs [75,76,77]. Transsynaptic signaling via brain derived neurotrophic factor and neurotrophin-3 has been shown to be crucial for the establishment of proper IHC innervation and neuronal survival [75,78,79,80,81] and the presence of DCVs at the presynaptic AZ early in development may hence suggest a role during the critical period of synapse formation and AZ refinement. 

Once the presynapse is structurally established, pre- and postsynapse undergo dramatic restructuring. During this transition, several seemingly essential presynaptic proteins—though initially expressed or at least detectable on mRNA level—are downregulated during IHC developmental maturation and finally entirely exchanged against IHC-specific molecules (Figure 4). In the following, we will now discuss this developmental transformation of the AZ and highlight a few established as well as more recently identified molecules relevant in this process.

### 2.3. Piccolino Acts as a Key Regulator of Ribbon Size and Shape

In IHCs, cytoplasmic ribbon precursors are commonly of spherical shape, highly heterogenous in size and already tether a set of SVs. Upon membrane attachment and functional maturation, the profiles of these immature precursors transition to a rather wedge- or droplet-shaped morphology [27]. Recently, a short C-terminally-truncated splice variant of Piccolo—termed ‘Piccolino’—was characterized that is specific for ribbon synapses of the eye and ear and can be found on cytoplasmically-floating precursors as well as membrane-attached ribbons alike [73,82]. In both, early postnatal and mature IHCs, Piccolino exerts a unique distribution pattern that appears to decorate the surface of cytoplasmically-floating as well as membrane-attached ribbon precursors [4]. Consistent with this localization, Piccolino is lost from presynaptic AZs upon genetic depletion of RIBEYE [18] but retains its colocalization with floating ribbons in *Bassoon* mutants [72]. These findings suggest that Piccolino is an integral part of the ribbon complex in IHCs. The functional role of Piccolino at the ribbon is likely to differ significantly from Piccolo function at conventional synapses, since its truncation causes Piccolino to be short of several interaction domains with other presynaptic proteins, including Bassoon, RIM2 and Ca_V_s. Yet, other interaction sites, for example, connecting Piccolino to the actin-based cytoskeleton as well as endocytosis-related proteins are retained [73]. KD experiments in murine photoreceptors suggest a function in the modulation of ribbon size and shape through scaffolding interactions with the RIBEYE B domain [33,82]. Therefore, ribbon morphology likely requires the presence of Piccolino as a multi-protein interaction hub, not only connecting to RIBEYE but also various other synaptic target proteins. Dependent on the respective KD efficiency, Piccolino-deficient ribbons were reported to be small-sized, membrane-attached spheres that failed to sustain adequate neurotransmission. Moreover, similar findings were reported from recently generated Piccolo gene trap rats [33], where the size alterations in rod photoreceptor ribbons coincided with a small—yet significant—reduction in synaptic transmission. Interestingly, this latter study further reported differential effects on photoreceptor as well as rod and cone bipolar cell ribbon shape, with not all cell types being affected to equal degrees. 

In the inner ear, the role of Piccolino remains elusive and the effect of Piccolino KD/KO is unknown to date. However, analogous to retinal ribbons, Piccolino is the dominant variant of Piccolo in IHCs [73,83]. The difference in ribbon morphology between retinal and auditory ribbons (i.e., IHC ribbons naturally are more spherical in shape; Figure 2), and even between the different retinal cell types, may therefore at least partially depend on the relative amounts of Piccolino or other, yet to be identified, cofactors present at the synapse. In such a scenario, distinct levels and/or protein ratios could modulate the ribbon shape to be more elongated and plate-like in the retina than in IHCs and vice versa [33]. In IHCs, Piccolino has been suggested to redistribute during developmental maturation and finally decorates the entire ribbon surface from hearing onset [4]—whether this distribution pattern is similar in developing photoreceptors remains to be investigated but may as well be involved in establishing a given ribbon morphology. Here, the molecular composition and expression levels of RIBEYE and other AZ proteins could co-determine the extent of shape regulation exerted by Piccolino. For example, upon Piccolino-KD in rod photoreceptors, not all ribbon shapes converted to spheres but a significant fraction of plate-shaped ribbons remained that merely decreased in height [82]. 

Apart from its apparent role in ribbon shape regulation, other specific functions of Piccolino remain to be demonstrated. Yet, experiments using an exogenous RIBEYE-binding peptide that spans the putative RIBEYE-Piccolino PxDLS-like interaction motif did not reveal any acute detrimental effects on SV release in retinal neurons, therefore seemingly arguing against an essential role of Piccolino in SV replenishment [21,33]. The same RIBEYE-binding peptide has also been extensively used in deciphering IHC presynaptic function but again, did not appear to exert any negative effects on SV exocytosis or replenishment [8,27,60,84]. However, the disruptions in the RIBEYE-Piccolino interactions may be too small or incomplete to acutely affect ribbon function.

In conclusion, although the exact function of Piccolino in IHCs remains elusive, (i) its early expression and localization to cytosolic ribbon precursors and (ii) the clear effects of attenuated expression on ribbon morphology and function in photoreceptors implicate Piccolino as a main contributor to IHC ribbon maturation and structural integrity.

### 2.4. Ribbon Anchoring at the AZ Membrane Critically Depends on the Large Cytomatrix Protein Bassoon

Upon their translocation to the AZ, ribbon precursors attach at the presynaptic membrane in a process involving the large scaffolding protein Bassoon [16,72]. Genetic deletion of Bassoon—in the cochlea and retina alike—prevents ribbon anchoring at the presynapse and ultimately leads to the loss and displacement of SVs from the AZ [8,16,72,85,86,87]. Hence, *Bassoon* mutants have previously been used as powerful tools to study ribbon function. In contrast to the majority of *Ribeye*-KO models, Bassoon-deficiency in IHCs heavily affects synaptic transmission, with a significant reduction in presynaptic Ca^2+^ currents and exocytosis of the RRP, finally leading to strongly impaired synchronous auditory signaling [8,16,85,87]. It seems that the remaining presence of the ribbons and thus the tethering and recruitment of SVs to the free-floating ribbons, prevented the developmental compensation occurring in *Ribeye*-KO models from being established in *Bassoon* mutant mice [8,16,18,27]. Alternatively, Bassoon may simply be strictly required for the establishment of presynaptic densities and Ca_V_ clustering. Interestingly in this context, ribbon loss from *Bassoon* mutant IHC AZs appears to be a progressive phenotype, where membrane-anchored ribbons are predominantly lost over the course of postnatal development [8,16,87]. Alongside ribbon detachment from the plasma membrane, Ca_V_ organization was severely compromised in this mutant and resulted in abnormal AZ clustering as well as diffuse presynaptic Ca^2+^ spread upon depolarization [8,69]. 

While the role of Bassoon in ribbon anchoring is well established, it still remains unclear when and how during developmental maturation it is transported to the AZ. In photoreceptors, Bassoon appears to travel as an integral part of the cytoplasmic ribbon precursors [88]; however, if this system is conserved in IHCs still awaits experimental confirmation. Alternative modes of transport may include dedicated dense-core granules—termed “Piccolo/Bassoon transport vesicles”—as has been shown in neurons [89] but again, their existence in IHCs remains to be unequivocally demonstrated. This task has however proven to be technically highly challenging [4]. Regarding the timing of arrival at the AZ, it is noteworthy that an electron-dense cytomatrix can already be observed at the presynaptic plasma membrane prior to ribbon precursor arrival [4,53,54]. Given Bassoon’s essential role in ribbon anchoring, it is hence tempting to speculate that Bassoon might present a core component of this meshwork.

One important remaining question is, whether the synaptic defects observed in *Bassoon* mutants are mainly illustrating the effects of ribbon loss or rather also reflect the lack of other essential Bassoon functions [8,16,86]. However, since the reduction in exocytosis was proportional to the loss of ribbons and dispersion of Ca_V_ clusters, one can argue that the missing ribbon-anchoring and scaffolding is the main effector [8,16]. Yet, a specific Bassoon function was revealed in a different *Bassoon* mutant with partially preserved interaction domains [8,87]. Here, ribbons remained present at the plasma membrane, but were anchored at a longer distance from the AZ membrane. Although this caused an intermediate phenotype in regards to Ca^2+^ currents and sustained exocytosis, the RRP and the reduced (spontaneous and evoked) SGN spike rate was similarly affected in both *Bassoon* mutants [87]. Thus, these findings support a scenario in which Bassoon may influence IHC exocytosis also directly, rather than exclusively via its role in ribbon anchoring. 

### 2.5. Establishing the Highly Efficient Ca^2+^-Dependent SV Release of Mature Ribbon Synapses

The optimization of Ca^2+^ dependent exocytosis is a hallmark of postnatal development of IHC AZs and an essential determinant of the exquisite temporal precision of the mature first auditory synapse. During this transformational process, extrasynaptic Ca_V_1.3 channels are eliminated from the basolateral plasma membrane and ultimately tightly clustered in a stripe-like organization below the ribbon. In parallel, the efficiency of excitation/secretion coupling is increased via functional maturation of the channel properties [8,16,26,27,29,45,50,90,91,92]. In addition, in the run-up to hearing onset, the spatial relation between Ca_V_s and release-ready SVs tightens from a micro- to a nanodomain-based coupling model ([27], reviewed in [93]). This refinement allows a single channel to orchestrate the release of a given SV, thereby ensuring utmost precision of signal transmission. Here, it is interesting to note that in cochlear hair cells, the expression of several spacer molecules of the Septin family that mediate SV/Ca_V_ spacing at, for example, the Calyx of Held synapse has been reported [94,95]. Moreover, some of the expression patterns appear to be developmentally regulated and thus might also be connected to this core aspect of maturational transformation [96]. AZs of mature IHCs have been shown to exhibit differential voltage-dependence of Ca^2+^ influx depending on their subcellular location [84], thereby hinting at a distinct molecular composition of individual AZs that may significantly contribute to synaptic heterogeneity and thus increase the dynamic range of sound encoding. How this functional gradient is molecularly regulated during postnatal maturation remains to be clarified, but appears to involve the intracellular trafficking molecule and deafness gene *Gipc3* [84]. 

Flawless regulation of Ca^2+^ influx can be considered an essential factor for correct synaptic maturation, maintenance and functional refinement [97,98,99] and appears to also modulate ribbon size during early development in zebrafish neuromast hair cells [100]. Thus, the mutation, genetic deletion or pharmacological inhibition of Ca_V_s can affect ribbon morphogenesis and occupancy at the AZ (Figure 4; [100,101]). Moreover, the KD of *ribeye a* [64] or *b* [9] in zebrafish caused a disconnect between the remaining ribbons and Ca_V_ clusters, which appear mislocalized in neuromast hair cells and diffuse in zebrafish retinal cells. Likewise, across all mammalian *Ribeye*-KO/KD models, the disorganization of Ca_V_s is a highly prevalent phenotype [9,17,18,19,64,65,66]. For example, instead of the typical stripe-like organization co-aligning with Bassoon, Ca_V_1.3 clusters are generally smaller and fragmented in *Ribeye*-KO mouse models [18,19]. Yet, overall Ca_V_ numbers do not appear to be affected by RIBEYE depletion and the observed mislocalization failed to compromise the SV/Ca_V_1.3 nanodomain coupling in mature IHCs [18,19], but led to an increased SV/Ca_V_1.4 distance at retinal bipolar to AII amacrine cell synapses [17]. Interestingly, RIBEYE depletion exerted subtle functional consequences on Ca_V_s in IHCs, including mildly enhanced Ca^2+^ current inactivation kinetics and a more depolarized voltage sensitivity [18], thereby again highlighting the intimate relationship between ribbons and presynaptic Ca_V_s. 

### 2.6. Developmental Transformation of the Ca^2+^ Sensing and Vesicular Fusion Machinery—An Acquired Key Role for Otoferlin

IHC presynapses are highly specialized structures that are molecularly distinct from conventional neuronal synapses. While many excellent reviews have summarized the findings on the molecular anatomy of mature IHC ribbon synapses (for example [10]), we will now take a closer look at the substantial structural and molecular re-organization of the IHC vesicular fusion machinery that occurs prior to hearing onset in mice—in particular during the first postnatal week. 

In IHCs, one of the key presynaptic molecules is Otoferlin, a large multi-C2-domain protein whose dysfunction underlies human non-syndromic recessive hearing impairment DFNB9 [102,103,104,105,106,107,108,109,110,111]. Otoferlin localizes to the presynaptic plasma membrane as well as to SVs, possesses multiple Ca^2+^- and phospholipid-binding sites and displays structural similarities to essential presynaptic proteins including Synaptotagmins (Syts) and Munc13s [107]. Thus, Otoferlin is thought to play a multi-faceted role in Ca^2+^ sensing, SV tethering and priming as well as RRP replenishment and in addition facilitates exo-/endocytosis coupling via its interactions with adaptor protein 2 (AP2) and Endophilins [22,108,112,113]. 

From a developmental perspective, several of these roles are of particular interest as they are acquired during a critical postnatal period of IHC maturation. In neurons and most other secretory cell types, Ca^2+^-sensing is performed by members of the Syt family—in particular Syt1 and Syt2. In mature IHCs, rather than Syts, Otoferlin appears to perform this task; however, vesicular Ca^2+^-sensing switches from an Otoferlin-independent to an Otoferlin-dependent mechanism around postnatal day (p)4 [114]. To date, the early ‘pre-Otoferlin’ Ca^2+^ sensor remains elusive and though initially suspected to be of the Syt family, various studies failed to detect major detrimental effects of genetic loss of Syt1, 2, 4 or 7 on the exocytic performance of newborn and/or adult IHCs [38,91,114,115]. While functional redundancy may—at least partly—compensate for the loss of individual isoforms and hence complicate the interpretation of these data, it is important to mention that the (temporal) expression patterns of the individual Syt isoforms in immature and mature IHCs still remain a controversial topic. Yet, specific roles of Syt1 in the recovery of the RRP in neonatal IHCs [114] and the non-Ca^2+^-binding Syt4 in conveying linearity to the Ca^2+^-dependence of SV release in mature IHCs [91] have been proposed. What remains unclear is why Otoferlin takes on this crucial task at such a relatively late postnatal stage and which other tasks this large multi-C2-domain protein may perform. Otoferlin can already be detected in IHCs as early as embryonic day (e)16 via immunohistochemical stainings, but reaches its maximum expression levels around (p)6 [105]—just prior to the peak Ca_V_ expression levels and maximum Ca^2+^ current amplitudes in developing IHCs [27,90,92]. Hence, this finding is seemingly compatible with the timeline of the observed developmental Ca^2+^-sensor switch. But what other role(s) might Otoferlin play in the preceding late embryonic/early postnatal development? Here, recent evidence from two separate studies, which aimed to gene-therapeutically restore Otoferlin-deficiency in *Otof*-KO mice, offer an intriguing new perspective: Otoferlin might actually also contribute to synapse assembly, maintenance and long-term structural integrity [116,117]. While IHC exocytosis and/or hearing could be restored in postnatally AAV-treated *Otof* mutants, both studies reported the characteristic decline in ribbon numbers with advancing age that was described to occur in *Otof*-KO mutants [105]. Hence, functional rescue after initial synapse assembly failed to reverse premature synapse loss in adulthood. If Otoferlin plays a direct or rather indirect role in this process will have to be clarified in future experiments.

In summary, these findings suggest a system in which Otoferlin plays a key role in the final steps of SV fusion and replenishment—in particular in IHC synaptic Ca^2+^-sensing, where it appears to be differentially supplemented by various Syt isoforms at distinct developmental time points and likely also contributes to the morphology and long-term structural integrity of IHC afferent synapses. Yet, how exactly Otoferlin conducts these various functions remains largely unclear to date. 

Apart from Syts, also other key constituents of the SV fusion machinery await definitive identification. While various in vitro assays point to a direct interaction of Otoferlin with Ca_V_s and members of the SNARE protein complex in a reconstituted system [102,103,104,118], cell physiological analyses of IHCs—after either clostridial neurotoxin treatment or genetic disruption—failed to reveal a functional role of Syntaxin-1/2/3, Synaptobrevin/VAMP-1/2/3 or SNAP25 [119]. Likewise, SNARE regulators such as complexins I-IV [120] and vesicular priming factors of the Munc13 and CAPS families appear to be absent from IHC AZs [108]. This latter finding is of particular interest since all other neuronal and neurosecretory systems—in invertebrates and vertebrates alike—and even other mammalian ribbon-type synapses in the retina and pineal gland [11,121,122], seem to employ these proteins to render SVs fusion competent (reviewed in Reference [123]). Therefore, future experiments will have to clarify which molecule(s) may substitute for Munc13 function in IHCs.

Nevertheless, it is worth mentioning that most constitutive KO mouse models for these essential synaptic proteins lead to early (neonatal) death of the mutant pups. Hence, several of the SNARE and all Munc13/CAPS mutants could only be analyzed after perinatal dissection and subsequent maturation under organotypic culture conditions. Thus, these findings may not adequately reflect synapse functionality in an intact and pre-/postnatally maturing system. To address these issues, future experiments will be required to employ conditional KO mutants for these genes once they become available and analyze the effects of acute genetic ablation once synapse assembly has concluded.

## 3. Structural Plasticity of Sensory Ribbon Synapses

In the second part of this review, we would like to summarize the current state of knowledge in regards to structural and functional plasticity of synaptic ribbons, ranging from developmental assembly to activity-modulation. While this latter phenomenon in particular remains largely elusive in cochlear hair cells, it is of utmost relevance in the quest to decipher ribbon function: Since ribbon size and SV tethering capacity are intimately linked, activity-dependent structural alterations may have a direct impact on the synaptic transfer function of individual AZs and could thus also contribute to the described functional heterogeneity observed within a single IHC [124]. Here, an example for the importance of this apparent structure-function relationship was reported in the retina, where the peak in size of photoreceptor ribbons was found to coincide with the lowest visual threshold in the dark state, thereby providing the greatest visual sensitivity [125]. Moreover, a separate study could show that activity-induced changes in ribbon size directly affect the clustering of other essential AZ components, including RIM2 and Ca_V_1.4 [66], therefore fundamentally changing the presynaptic composition based on the acute activity state.

In the following, we will now discuss the available data on this topic in more detail—with a particular focus on the mammalian retina and zebrafish lateral line. Here, a picture emerges in which ribbons are indeed capable of dynamically adapting their size, shape, number per AZ as well as localization during developmental maturation and in response to different states of activity [4,11,66,74,126]. Finally, we will infer potential molecular pathways through which structural plasticity of IHC ribbons—during development but also in response to activity modulation—could be conveyed.

### 3.1. Ribbons Are Intrinsically Dynamic Scaffolds

Structural plasticity requires a long-term stable—yet adaptive—scaffold that is capable of undergoing morphological changes in response to a recurrent stimulus. In zebrafish, it could be shown that within a given membrane-anchored ribbon, Ribeye molecules exert a high degree of mobility and turnover [126,127], an important feature ensuring structural maintenance over extended periods of time. Interestingly, within the structural context of a ribbon, Ribeye molecules were shown to be less mobile and their turnover slowed in comparison to cytoplasmic Ribeye aggregates lacking synaptic context [127]. Thereby, the multi-protein framework of the ribbon appears to act as a stabilizing scaffold effectively ‘trapping’ otherwise highly mobile Ribeye molecules, while allowing continuous intra-structural protein turnover. 

Moreover, at murine retinal bipolar cell ribbons, it could be shown that the juxtaposition of a presynaptic ribbon to a postsynaptic density (PSD) stabilized the synaptic complex as a whole, since membrane-proximal RIBEYE aggregates lacking a PSD were disassembled more rapidly than synaptically-engaged ones [67]. To date, little is known about these processes in cochlear IHCs and intra-ribbon protein turnover and structural maintenance remain topics of intense research.

### 3.2. Structural Plasticity during Presynaptic Assembly and AZ Maturation

As discussed above, the developmental maturation of IHC presynapses is a highly plastic process, which is characterized by the streamlining of complex multi-ribbon AZs towards predominantly single ribbon-bearing synaptic contacts after hearing onset [4,27]. The underlying pathways facilitating this refinement process appear to be mainly based on multi-ribbon fusion rather than selective degradation of non-dominant ribbons, since the ribbon-occupied volume per AZ remains comparable between premature and mature synaptic contacts [4]. One interesting aspect in this regard is the fact that multi-ribbon AZs can still be detected in adult IHCs as well as pinealocytes and photoreceptors [4,128,129]. This latter observation raises the fundamental question if a subset of multi-ribbon AZs is maintained from development to adulthood (e.g., dependent on the subcellular localization of the synapse, etc.) and further, how—despite of their apparent fusogenicity—some ribbons are kept in close proximity, but yet remain separate entities. A rather simple explanation here may include the degradation of an ‘old’ ribbon scaffold, while a new ribbon is being built within direct proximity to retain functionality of the respective release site. Here, the observation of ‘hollow’ ribbons, that is, ribbons with a translucent core in EM micrographs—has been proposed to signify presynaptic scaffolds undergoing degradation [4,53,55]; however, the current information obtained from static imaging is unsuitable to fully clarify this issue. An alternative explanation in this context may involve the differential expression of molecular spacers that could form a physical barrier preventing ribbon fusion. As already mentioned above, at neuronal synapses, such scaffold proteins have been shown to create structural and functional distance between presynaptic components including SVs and presynaptic Ca_V_s [94,95]. Likewise, such a mechanism could also contribute to the spatial separation of multiple ribbons at a single AZ. However, while the mRNAs of various Septin isoforms can be detected in developing IHCs [96], genetic deletion of Septin-4 and -5 did not affect auditory performance in the respective mutants and protein expression appeared rather limited to supporting cell types and efferent presynaptic terminals [130]. Therefore, involvement of Septin isoforms and/or other molecular spacers in this process or SV/Ca_V_-coupling remains to be demonstrated.

Once assembled, synaptic maintenance and ribbon growth does not solely depend on ribbon precursor fusion at or in close proximity to the AZ. Rather, recent work on zebrafish neuromast hair cells suggests that a cytoplasmic pool of Ribeye molecules facilitates protein turnover within membrane-anchored ribbons [126]. These individual molecules may also ‘precipitate’ on existing ribbon scaffolds, thereby likely assisting in ribbon growth in addition to precursor fusion. Intriguingly, in the same study, experiments involving fluorescence-recovery after photobleaching (FRAP) of individual ribbons suggest that synaptic components might be readily exchanged between proximal ribbons, since photobleaching of a single ribbon also mildly reduced the fluorescence intensity of the other ribbons within an individual hair cell. Here, the underlying mechanisms may include diffusional exchange via the cytoplasmic pool of Ribeye molecules or a targeted exchange of synaptic components via a more sophisticated—so far undescribed—inter-synaptic transport system. Whether such mechanisms also operate in mammalian IHCs remains to be shown.

### 3.3. Activity-Dependent Structural Plasticity of Ribbon-Type AZs

The most dramatic phase of AZ re-shaping coincides with the peak of spontaneous activity in the organ of Corti. These randomly but regularly occurring Ca^2+^ spikes trigger synaptic release events in developing hair cells that are essential for the refinement of the tonotopic organization of the auditory pathway and circuit maturation [92,131,132,133,134,135,136]. While this pre-sensory neurotransmission does not appear to be essential for initial synapse assembly and medium-term structural maintenance [98,137], it could recently be shown to play a critical role in driving postsynaptic SGN subtype diversification [138,139] and promoting neuronal survival [140]. It is hence conceivable that it may also present a main determinant of presynaptic AZ functional heterogeneity and contribute to the establishment of ribbon size gradients [8,84,128]. 

**Figure 5:**
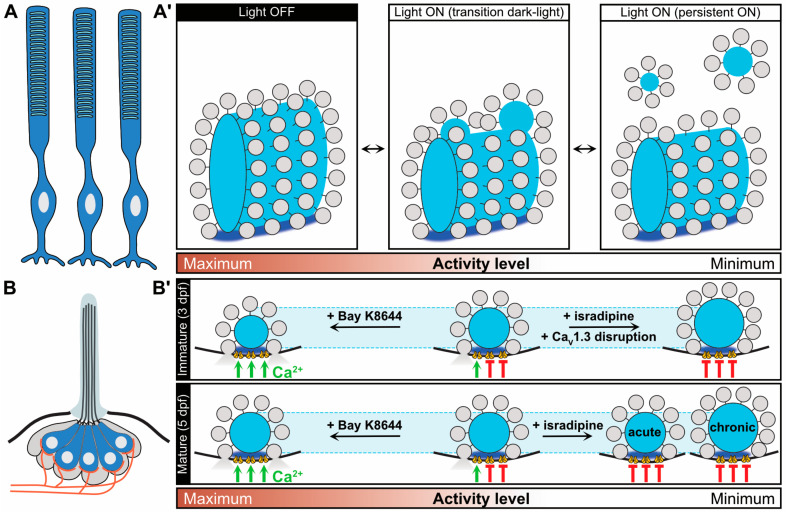
Presynaptic structural plasticity at sensory ribbon synapses. (**A**) In retinal photoreceptors, ribbons can reversibly adjust in size upon exposure to light. (**A’**) Light-induced plasticity includes the detachment of spherical aggregates from the ribbon apex that continue to tether synaptic vesicles (SVs) and, upon prolonged light exposure, become free-floating in the cytoplasm. As a consequence, the membrane attached ribbon is smaller in the light phase than in the dark phase. When transitioning back to darkness, the ribbon regains material and rebuilds its size. Thereby, the largest ribbon size—thus largest SV tethering capacity—is coinciding with the highest activity state of photoreceptors during the dark phase. (**B**) In zebrafish lateral line neuromast hair cells, ribbons can show plastic changes in response to different levels of Ca^2+^ influx. (**B’**) Here, the prolonged opening of presynaptic Ca_V_s by BayK8644 application during early development (3 days post-fertilization, dpf) causes a decrease in ribbon size (upper panel), while the disruption of Ca_V_ function, either by genetic manipulation or isradipine treatment, causes an increase in ribbon size. Remarkably, in more matured hair cells (5 dpf; lower panel) the ribbons are unaffected by acute BayK8644 treatment, nevertheless ribbon size enhancement could be induced by long-term isradipine treatment. In direct contrast to the mammalian retina, the largest ribbon size—and hence largest vesicle pool—coincides with the lowest level of Ca^2+^ influx.

While IHC ribbons are evidently structurally plastic during early postnatal development, activity-dependent plasticity of cochlear ribbons—during developmental maturation as well as in adulthood—remains largely elusive. However, in various ribbon-containing retinal cell types as well as pinealocytes, morphological adaptations in response to synaptic activity have previously been documented (reviewed in Reference [129]; Figure 5). For example, changes in ribbon size, shape, number and structural density as well as association with multiple AZ proteins have been described in response to different states of activity, for example, upon (i) changes in illumination [66,74,125,141,142,143,144], (ii) the diurnal cycle [11,56,145,146,147,148] and (iii) hibernation [71,149]. Yet, this topic still remains somewhat controversial, in particular in non-mammalian species. In mammals on the contrary, the available information seems more consistent—although mouse strain differences have been reported [141].

#### 3.3.1. Presynaptic Ca^2+^ Influx Is a Key Regulator of Ribbon Size and Structural Integrity

Mammalian photoreceptor ribbons have been shown to increase in size during darkness and rapidly decrease in size upon light stimulation [56,66,74,125,141,145]. During the light phase, ribbon material appears to detach from the ribbon in spherical clusters via protrusion at the membrane-distal part of the plate-shaped ribbon. Upon darkness, the free-floating ribbon spheres seemingly re-fuse with the membrane-anchored ribbons, thus again increasing ribbon size (Figure 5A,A’; [74]). Remarkably, cytosolic ribbon spheres appeared to mostly merge with the side of the ribbon, thereby creating activity-dependent turnover of ribbon material. Extracellular application of the Ca^2+^ chelators EGTA and BAPTA to reduce synaptic Ca^2+^ influx has been used to simulate photoreceptor hyperpolarization upon illumination in vitro and caused a similar decrease in ribbon size, as well as a significantly higher fraction of free-floating ribbon spheres. Long-term exposure to EGTA—which effectively silences the release site—eventually dispersed the presynaptic complex and revealed a two-step process in which ribbons degrade first and other presynaptic AZ components appear to be initially more resilient [74,88]. Conversely, an increase in intracellular Ca^2+^ levels could reverse the light-induced effects, mirroring the enhanced activation of the photoreceptors in the dark [74]. Therefore, the presynaptic Ca^2+^ concentration appears to be a key determinant of ribbon size in photoreceptors.

Similar to photoreceptors, pinealocyte ribbons have been shown to also reversibly increase in size as well as number overnight and structurally re-organize from a plate- to a horseshoe-like shape [11,34,146,150,151]. In this process, stimulatory hypothalamic input to the pineal gland has proven essential, as ribbon dynamics were absent when noradrenergic input was blocked [11]. Interestingly, this structural plasticity was further characterized by the recruitment of the AZ proteins Bassoon, RIM2 and Munc-13 to the ribbon during the night and an opposing preference for the clustering of molecular motor subunit Kif3a during the day.

#### 3.3.2. Tight Presynaptic Ca^2+^ Regulation Is Essential for Ribbon Assembly in Zebrafish Neuromast Hair Cells

Analogous to these mammalian preparations, in fish photoreceptors [142] and zebrafish neuromast hair cells [9,35,100], presynaptic Ca^2+^ levels have also been shown to regulate ribbon morphology, especially during ribbon synapse assembly (Figure 5B,B’). Here however, pharmacological inhibition and genetic inactivation of Ca_V_s during early development caused (i) ribbon enlargement, (ii) an increase in ribbon number per AZ, (iii) increased cytosolic ribbon counts and (iv) the appearance of odd-shaped ribbons [100]. Consistent with these observations, enhanced Ca^2+^ influx decreased ribbon size and number in these experiments. Hence, presynaptic Ca^2+^ influx, clustering of Ca_V_s and regulation of ribbon size are closely connected and dynamically changing Ca^2+^ levels—triggered by for example, spontaneous activity during maturation—likely serve an important function in ribbon refinement and structural plasticity. Moreover, in the described regulatory link between presynaptic Ca^2+^ levels and ribbon morphology in zebrafish, an important role is played by synaptic mitochondria, which have been shown to localize in close proximity to synaptic ribbons in lateral line neuromast hair cells [152], mammalian IHCs [153,154] as well as vestibular hair cells [155]. During development, spontaneous activity-driven presynaptic Ca^2+^ influx initiates mitochondrial Ca^2+^ uptake in the presynaptic region in neuromast hair cells—and likely also IHCs (though this still needs to be demonstrated)—which in turn attenuates the local NAD^+^/NADH ratio in direct ribbon proximity [152]. Since RIBEYE has been shown to contain a putative NAD(H) binding site [6,58], such differential redox regulation may alter intra- and intermolecular RIBEYE-RIBEYE interactions, thereby directly contributing to morphological plasticity of the ribbon scaffold. Indeed, in neuromast hair cells, alterations of the synaptic redox state were recently shown to dynamically modulate—and ultimately restrict—ribbon size during maturation [152]. 

#### 3.3.3. Mature Ribbons Are Structurally More Stable Scaffolds Than Developing Ribbons

In the context of activity-dependent ribbon size regulation, it is of particular interest to now also compare the differential plasticity of developing and mature ribbons. Over the course of postnatal maturation, the synaptic ribbon advances to form a seemingly stable structure; however, even mature ribbons should not be considered as entirely static and rigid scaffolds. For example, more matured zebrafish neuromast hair cell ribbons (5 days post-fertilization; dpf5) proved to be less sensitive to pharmacological Ca_V_ inhibition than the ones from younger cells (dpf3) and required significantly longer treatment to trigger structural plasticity of the ribbon scaffold (Figure 5B’; [100]). While this latter finding suggests that mature ribbons are generally more stable structures than developing ribbons, it also indicates that ribbon shape—and the AZ molecular composition in general—might still be modulated by the state of activity of a given AZ, even in adult tissue. Similar observations have also been made in mammalian preparations, where activity manipulation in mice exerted significant effects on mature ribbon synapse architecture [156,157]. For example, prolonged deprivation of auditory input during the final stages of synapse maturation led to marked alterations in ribbon size alongside an upregulation of the molecular components of the postsynaptic compartment—including AMPA receptor subunits and the postsynaptic density protein Shank1 [156]. This putative compensatory upregulation did however not affect synapse number and the physiological implications of this adaptation remain to be determined. Remarkably, in this study the postsynaptic alterations preceded the presynaptic increase in ribbon size by a week, a finding that may suggest the involvement of retrograde transsynaptic signaling pathways in this process (reviewed in Reference [158]). Similarly, directly after short-term acoustic overexposure, ribbon volume is significantly increased [157], again arguing for a highly adaptive presynaptic complex that might be driven either by direct activation or retrograde neuronal signal transduction.

#### 3.3.4. Evidence for Ribbon Mobility at the AZ Membrane

Finally, next to the discussed structural plasticity of presynaptic ribbons in response to altered activity levels, also ribbon *positional* plasticity at hair cell AZs might have to be considered. Here, live-cell imaging data zebrafish neuromast hair cells offer an intriguing new perspective: Supposedly synaptically-engaged ribbons appeared to ‘drift’ at the presynaptic plasma membrane, switching between periods of higher mobility and greater stability [126]. This is a particularly interesting finding, since it may have additional implications for release efficiency of a given synaptic contact. Thus, it would be of great interest to identify if these bouts of increased or decreased mobility also occur at IHC AZs, differ depending on the subcellular localization of the respective synapse (i.e. modiolar vs. pillar ribbons) and are synchronized with synaptic activity levels. Interestingly, recent data from acoustic overexposure experiments suggest that afferent synapses within the synaptopathic region transiently disperse along the habenular/cuticular IHC axis during noise exposure, but appear to be re-confined to the basolateral pole within a week of recovery [157]. Again, these data are suggestive of a rather fluid anchoring system of the (pre-)synaptic complex. Yet, the molecular mechanisms driving this reversible structural dispersion require further investigations.

### 3.4. Molecular Pathways Involved in Ribbon-Type AZ Assembly and Structural Plasticity

In this final section, we will discuss how ribbon precursor transport to—as well as precursor fusion at—the AZ membrane may be achieved and how ribbon structural plasticity may be regulated on the molecular scale. 

**Figure 6:**
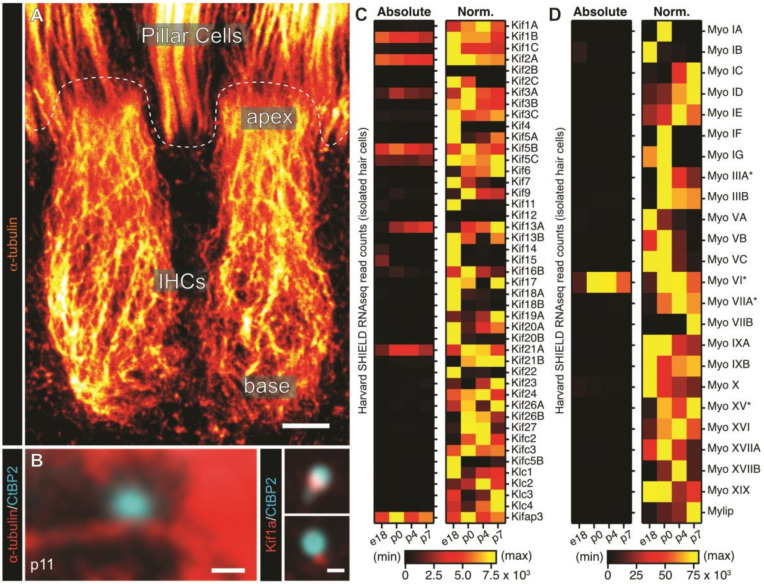
The role of cytoskeletal transport in IHC presynaptic AZ assembly. (**A**) The longitudinal microtubule network of murine inner hair cells (IHCs) is polarized in an apico-basal orientation. Shown is a super-resolution (STED) maximum projection of two p14 IHCs labeled against α-tubulin visualized with an intensity-coded look-up table, where warmer colors indicate higher intensities. (**B**) Representative STED images of cytoplasmically free-floating ribbon precursors (cyan) are located in close proximity to microtubule tracks (red) in IHCs prior to hearing onset (left panel) and colocalize with microtubule-based motor Kif1a (right panels). This suggests microtubule-based transport of ribbon material during development. (**C**,**D**) Developmental expression patterns of Kinesin motors (**C**) and Myosin motors (**D**), based on publicly-available RNA sequencing data of isolated murine IHCs replotted from the SHIELD database ([96]; https://shield.hms.harvard.edu/index.html). Illustrated are absolute expression patterns from embryonic day (e)18 to postnatal day (p)7 and the same data normalized to their maximum expression level over this time period to reveal temporal expression patterns of the individual targets. (*) indicates motor proteins linked with hereditary syndromic and/or non-syndromic hearing loss in humans (according to https://hereditaryhearingloss.org; October 2020). Scale bars: A 2.5 µm; B left panel: 250 µm, right panels: 200 µm. (**B**) with permission from Reference [4].

Targeted trafficking systems commonly utilize the cellular cytoskeleton, where long-distance transport is achieved via microtubule (MT)-based transport and short-distance peripheral distribution along the plasma membrane rather employs the cortical actin meshwork [159]. While the exact cytoskeletal components and molecular motors responsible for IHC ribbon precursor transport and structural plasticity have remained largely elusive so far, potential candidate molecules can be identified from (i) immunolocalization experiments [4], (ii) studies on mutant mice and/or pharmacological manipulation experiments [160,161,162,163] and (iii) publicly-available single-cell RNA-sequencing databases, such as the Shared Harvard Inner-Ear Laboratory Database (SHIELD) (https://shield.hms.harvard.edu/index.html [96,164]), gEAR (https://umgear.org) and others. Here, a plethora of molecular motors with differential temporal expression patterns can be identified that show affinity for AZ proteins, have been implicated in SV or organelle transport at conventional synapses, and/or are linked to genetic hearing loss and hence might (also) contribute to ribbon dynamics (Figure 6).

### 3.5. Microtubule-Based Trafficking of Ribbon Precursors to the AZ

MT bundles form essential apico-basal transport pathways reaching all the way from the cuticular plate to the presynaptic compartment (Figure 6A; [165,166]). These long-distance transport hubs have therefore been implicated in IHC presynaptogenesis [4], although early reports on the affinity of ribbons for MTs have been contradictory [167,168]. Biochemical analyses of isolated ribbon synapse fractions indicated a direct association and (partial) colocalization of the ribbon with both, actin and tubulin and thus may corroborate the involvement of cytoskeletal tracks in ribbon transport to the AZ [169]; however, as also noted by the authors, potential contamination of the isolated synaptic fractions—in particular by actin-dense stereocilia—could not be excluded in these experiments. Yet, consistent with such a hypothesis, recent findings on AZ-directed ribbon precursor transport have provided further evidence for a MT-based pathway in IHCs, specifically by involvement of MT plus-end directed Kinesin motor proteins [4]. Here, in particular, the Kinesin III-type monomeric motor Kif1a was found to colocalize with cytosolic ribbon precursors in developing IHCs and the measured nearest-distance between precursor-attached SVs and proximal MTs was consistent with the suspected length of Kif1a motors (Figure 6B; [4,170]). Considering the role of Kif1a in SV precursor transport at conventional synapses [171,172,173], these findings suggest Kif1a to contribute to AZ-targeting ribbon transport in IHCs. Indeed, a role of MT plus end directed motors such as Kif1a seemingly fits into the larger picture: in polarized epithelial cells the MT orientation is largely determined by the position of the centriole, which anchors the MT minus ends and is located below the cuticular plate in IHCs [174]. Therefore, the predominant MT growth direction can be assumed to extend from the apex of the cell towards the base [174,175]. Apart from Kif1a, two other members of the plus end-directed Kinesin family are known to be involved in AZ protein transport, namely Kinesin I motor complex Kif5 and Kinesin XIII component Kif2a [176,177]. However, while both are indeed expressed in IHCs [96], they failed to colocalize with ribbon precursors [4].

One other controversially-discussed Kinesin that may be involved in the transport of IHC ribbon precursors and possibly SVs, is the moderately expressed Kinesin II motor Kif3a [11,62,96]. In photoreceptors, Kif3a colocalizes with the ribbon and SVs [11,62,72] and the temporal ribbon-association pattern of Kif3a has been shown to mirror the diurnal ribbon cycle in pinealocytes, where it strongly associates with the ribbon during the daytime, but is largely absent during the night [11]. Because of the plus-end directed movement of the Kinesin II motor family, aggregation of Kif3a on one specific side of the ribbon could indicate accumulation at the end of—or detachment from—MT tracks. This would suggest the transport of ribbon or SV material—possibly to replenish and re-establish the ribbon—in the counterintuitive direction, since pinealocyte ribbons in fact loose material during the daytime and regain size at night. Here, high rates of transport of ribbon material during the night may lead to accumulation of Kif3a in the morning, which is then slowly removed during the course of the day. 

Genetic loss of Kif3a in cochlear hair cells causes severe disruptions in hair bundle morphology and defects in the establishment of planar cell polarity [178]. This compromises sensory input but may thereby also affect MT organization, because of centriole defects and basal body misplacement on both, planar and apico-basal axes. While in-depth analysis of the presynaptic AZ is still lacking in these mutants, it is worth mentioning that retinal depletion of Kif3a did not appear to affect ribbon morphology or function [179]. 

To date, while a plethora of Kinesins is expressed and differentially regulated during cochlear hair cell developmental maturation (Figure 6C)—to our knowledge—no genetic mutations in Kinesin genes have so far been implicated in IHC synapse assembly and maintenance or human hearing loss.

### 3.6. A Putative Role of Actin in Ribbon Assembly and IHC Exocytosis

Apart from MTs, hair cells express a dense sub-membranous actin cytoskeleton, the so-called *cortical lattice*, which has been proposed to mainly consist of a densely packed actin meshwork connected by various Spectrin isoforms [180,181,182]. At the AZ, synaptic ribbons localize in close proximity to the actin network and, as mentioned above, may (more or less directly) interact with the actin filaments [169]. Pharmacological depolymerization of the actin cytoskeleton in mature IHCs disorganized and scattered Ca_V_1.3 clusters and simultaneously resulted in a facilitation of exocytosis [162,163]. While Ca^2+^ currents were unaffected, the enhanced exocytosis could be reversed by EGTA application, thereby suggesting an interdependence of presynaptic Ca^2+^ channels, SVs and subcortical actin at the presynaptic membrane [162]. The authors proposed several explanations for the observed enhancement of exocytosis, stating that actin depolymerization could (i) compromise the actin-based ‘fusion barrier’ (i.e. a cortical impediment preventing SVs from fusing with the plasma membrane outside the dedicated release sites), (ii) lead to an increase in osmotic pressure and a concomitant increase in exocytosis, (iii) alter the replenishment of SVs or (iv) result in an altered SV/Ca_V_ coupling distance [162,163]. Here, the latter hypothesis is specifically interesting, considering the above-mentioned developmental transition from micro- to nanodomain coupling between SVs and Ca_V_ that takes place prior to hearing onset. If actin is indeed a contributing factor in the maturational tightening of the SV/Ca_V_ coupling distance remains to be clarified.

Apart from its described roles in membrane scaffolding and IHC exocytosis, it is tempting to speculate that the dense cortical actin meshwork might additionally serve a transport function, mediating exchange of synaptic material between adjacent AZs; however, data supporting such a scenario is lacking to date.

A multitude of actin-based molecular motors have been identified in hair cells that, similar to Kinesins, show differential protein expression patterns during hair cell development (Figure 6D; [183]). In particular, unconventional Myosins—such as Myosin VI among others—have been attributed essential roles in hair bundle morphogenesis, maintenance and mechanotransduction (reviewed in Reference [184]). However, some of these, such as Myosin Va [161] and Myosin VI [160], have also been linked to ribbon synapse development in photoreceptors and IHCs respectively. 

Myosin Va has previously been proposed to play a role in photoreceptor ribbon morphology and maintenance of function [161]. Here, depletion of Myosin Va affected synaptic transmission and induced the deformation of ribbons, albeit in only a small percentage of cells (~5%). Remarkably, ribbon shape in Myosin Va-deficient cells was more spherical and club-like, resembling the ribbons involved in illumination-induced plasticity [74,161]. Myosin Va may thus contribute to the proper assembly and/or structural plasticity of mature plate-like photoreceptor ribbons. Moreover, the colocalization of Myosin Va with ribbon-tethered SVs and the misplacement of SVs upon loss of Myosin Va, may suggest an additional function in the association of ribbons and SVs [161]. Interestingly, Myosin Va has been linked to the MT-based motor Kif5a, serving as a complex to transfer cargo from MTs to actin filaments [177]. This could point to potential interplay of the two cytoskeletons in ribbon plasticity or possibly ribbon-SV association. 

Myosin VI is highly expressed in cochlear hair cells and has previously been implicated in hair bundle morphogenesis [185,186,187,188], mechanotransduction [189] and ribbon synapse maturation [160]. Genetic disruption of Myosin VI in IHCs interfered with the postnatal development in mice, causing a severe underdevelopment phenotype at hearing onset that persisted into adulthood in a subset of IHCs [160,185,187]. Myosin VI depletion reduced exocytosis, diminished release efficacy and induced the persistent presence of morphologically immature ribbons as well as general ribbon loss [160]. Whether Myosin VI directly regulates ribbon dynamics or if the displayed mutant phenotype rather reflects downstream consequences of the pronounced hair bundle defects remains unclear to date. Since the dysfunctional Myosin VI phenotype only appears after the establishment of Otoferlin-dependent exocytosis [114], the authors speculated that an involvement of Myosin VI in ribbon maturation may be exerted via its direct interaction with Otoferlin [112,190]. In such a scenario, the delayed maturation of exocytosis may indirectly affect ribbon morphology. 

To date, a range of mutations in Myosin-encoding genes could be linked to genetic hearing loss (Figure 6D). For example, Myosin VI-deficiency underlies the autosomal as well as recessive non-syndromic forms of hereditary hearing impairment DFNA22 and DFNB37 [191,192]. Myosin VIIa mutations cause Usher syndrome USH1B [193,194,195] and are also implicated in non-syndromic DFNA11 and DFNB2 [196,197]. Similarly, Myosin XVa, a stereociliar elongation factor (DFNB3, [198]) and Myosin IIIa, which contributes to hair bundle morphogenesis (DFNB30, [199,200]), have also been identified in this context. This strong correlation between Myosin dysfunction and peripheral hearing deficits, plus the still unresolved role of multiple Myosins in certain aspects of IHC physiology, may thus indicate an involvement of these motor proteins in synaptic sound encoding, which might—at least in part—also result from corrupted presynaptogenesis. Yet, the severe hair bundle defects observed in several Myosin mutants make interpretation of loss-of-function mutations exceedingly difficult and illustrate the importance of intact cellular transport pathways for overall hair cell development. 

Next to the discussed effects on ribbon morphology, it is worth mentioning that—by analogy to conventional neuronal synapses—both Myosin V and Myosin VI likely exert additional roles in organelle transport as well as SV exo-/endocytosis that can be inferred but remain to be confirmed in IHCs [201,202,203,204].

In summary, multiple pathways appear to contribute to IHC ribbon development and structural plasticity, likely involving both, the MT as well as the actin cytoskeleton. The identification of ribbon-regulating molecular motors will be a major task of future studies, for which the SHIELD database (Figure 6C,D; [96,164]) and other single-cell sequencing platforms should form a starting point. In addition to the discussed candidates, various other Kinesins and unconventional Myosins are expressed in IHCs but their potential involvement in ribbon transport remains to be determined. Here, examples include Myosin IXa, Myosin XVI or Kif13a and so forth [170]. Furthermore, future studies will also have to investigate the expression and functional role of retrograde motors, such as MT minus-end directed Dyneins. For example, the gradual increase in expression of Dynein L8 light chain 2 during IHC development, plus its link to AZ protein transport, make for another likely candidate involved in ribbon plasticity regulation [96,205]. Finally, detailed localization and live-cell imaging studies will be required to determine the main molecular motors involved in ribbon precursor transport and putative inter-synaptic exchange of ribbon material.

## 4. Conclusions and Perspectives

Ribbon synapse assembly and presynaptic structural plasticity in cochlear IHCs remain enigmatic topics in hair cell development and synaptic functionality. While vast progress has been made over recent years in determining the molecular composition and function of mature ribbons, the regulation of their synthesis, transport to the AZ and molecular maturation are still largely unclear. Upon initial assembly, IHC ribbon synapses undergo a dramatic structural and molecular refinement to optimize the efficiency of glutamate release and synaptic signal transfer [4,27,46,53]. This refinement ranges from (i) a structural confinement of multi-ribbon AZs and extrasynaptic Ca_V_s and (ii) the tightening of the SV-Ca_V_ relationship to (iii) a complete replacement of the vesicular Ca^2+^-sensor within the matter of a few days during early postnatal development. Currently, the underlying factors and signaling pathways determining this remarkable transition remain to be identified. Another major area that requires a deeper understanding is the hair cell cytoskeletal transport system and—in particular—its role in ribbon assembly but also structural plasticity and inter-synaptic exchange of synaptic components. SV-bearing ribbon precursors can be observed floating in the cytoplasm from the late embryonic stages and have already been described in early morphological studies in the early 1980’s [53]; yet, even today we still do not know how exactly these complex multi-protein organelles are formed and we are just slowly beginning to understand which molecular transport pathways they might utilize to translocate to the AZ. Moreover, apart from a critical role of thyroid hormone in cochlear maturation, it is still largely unknown, which molecules and intracellular signaling pathways facilitate AZ formation at initial afferent contacts with SGNs and which process determines contact survival and synaptic maturation throughout the maturational pruning phase of IHC-SGN contact sites [46,50,51].

Thus, several important questions remain in this field, which will have to be addressed in future experiments. These may include: What is the molecular composition of auditory ribbon precursors? Which factors facilitate/prevent ribbon fusion at the AZ? How structurally adaptive are individual auditory ribbons before and after hearing onset—in particular on short to medium timescales (i. e., minutes–hours)—and what impact might this have on SV release capacity of a given synaptic contact? Is there bulk exchange of synaptic material between adjacent IHC AZs/ribbons? If so, which cytoskeletal components and molecular motors are involved? Does persistent/recurrent synaptic activity (spontaneous during developmental maturation or sound-induced in adulthood) favor certain AZs based on their cellular location and hence drive or at least contribute to presynaptic molecular and/or structural heterogeneity? 

All these questions will need to be addressed in future work. Fortunately, recent advances in microscopy—such as the wider availability of super-resolution techniques—in combination with the application of genetic and especially optogenetic tools, will allow the development of novel experimental approaches to clarify these issues.

## Figures and Tables

**Table 1:** Ribbon dimensions in different sensory cell types and species.

Cell Type	Species	Profile Shape	Ribbon			References
			Height (nm)	Length (nm)	Width (nm)	
**Inner ear**						
IHCs	mouse	wedge/droplet	100–500	~190	~120	[4,18,26,27]
**Retina**						
Photoreceptors (Rod) (Cone)						
	mouse	plate	150–400	30–40	500–1800	[28,29]
	mouse	plate	150–350	30–60	200–700	[30,31]
Bipolar cells (Rod)	mouse	plate	125-180	~50	~175	[32,33]
**Pineal gland**						
Pinealocytes	rat	plate	200–400	30–40	~800	[34]
**Lateral line**						
Neuromast HCs	zebrafish	sphere	200–400	200–400	200–400	[35,36]

All data derived from electron microscopic analysis.

## Abbreviations

AZ	Active zone
Ca_V_	Voltage-dependent Ca^2+^ channel
DCV	Dense-core vesicle
EM	Electron microscopy
IHC	Inner hair cell
KD/KO	Knock-down/-out
MT	Microtubule
OHC	Outer hair cell
PSD	Postsynaptic density
RRP	Readily-releasable pool
SGN	Spiral ganglion neuron
SV	Synaptic vesicle
